# Retraction Note: The interrelationships between Chinese learners’ trait emotional intelligence and teachers’ emotional support in learners’ engagement

**DOI:** 10.1186/s40359-025-02816-8

**Published:** 2025-05-09

**Authors:** Yao Yan, Xusheng Zhang, Tong Lei, Pei Zheng, Chao Jiang

**Affiliations:** 1 School of New Media Art, Xi’an Polytechnic University, Xi’an, 710048 China; 2 College of Art, Anhui University of Finance and Economics, Bengbu, 233000 China

**Retraction Note: BMC Psychology (2024) 12:35**

10.1186/s40359-024-01519-w

The editor and the publisher have retracted this article. The article was submitted to be part of a guest-edited issue. An investigation by the publisher found a number of articles, including this one, with a number of concerns, including but not limited to compromised editorial handling and peer review process, inappropriate or irrelevant references or not being in scope of the journal or guest-edited issue. Based on the investigation’s findings the editor therefore no longer has confidence in the results and conclusions of this article.

The authors have not responded to correspondence regarding this retraction.

